# Long-Term Prednisone Use Increases Hepatocellular Carcinoma Risk in Autoimmune Hepatitis Cirrhosis: A Retrospective Cohort Study

**DOI:** 10.1016/j.gastha.2025.100784

**Published:** 2025-08-30

**Authors:** Jessica Liu, Rami Hemadeh, Abdelrahman M. Attia, Pojsakorn Danpanichkul, Hasmik Adetyan, Naomy Kim, Tamar Yalda, Ju Dong Yang, Manaf Alsudaney, Walid Ayoub

**Affiliations:** 1 Chicago Medical School, Rosalind Franklin University of Medicine and Science, North Chicago, Illinois; 2 Department of Medicine, Karsh Division of Gastroenterology and Hepatology, Cedars-Sinai Medical Center, Los Angeles, California; 3 Comprehensive Transplant Center, Cedars-Sinai Medical Center, Los Angeles, California; 4 Department of Internal Medicine, Texas Tech University Health Sciences Center, Lubbock, Texas; 5 Samuel Oschin Comprehensive Cancer Institute, Cedars-Sinai Medical Center, Los Angeles, California

**Keywords:** Prednisone, Hepatocellular Carcinoma, Autoimmune Hepatitis, Cirrhosis

## Abstract

**Background and Aims:**

Autoimmune hepatitis (AIH) is an immune-mediated liver condition marked by progressive inflammation, piecemeal necrosis, and eventual cirrhosis. Prednisone has long served as the foundational AIH therapy. However, its prolonged use warrants closer evaluation regarding long-term outcomes. Among cirrhotic patients, the combination of chronic inflammatory and tumor-promoting changes may synergize to compromise immunity increasing susceptibility to hepatocellular carcinoma (HCC). In this context, adding immunosuppressive burden of corticosteroids may influence cancer risk in ways not well investigated. We investigated the effect of dose-dependent prednisone exposure on HCC risk in AIH-cirrhosis patients.

**Methods:**

We conducted a retrospective analysis of 121 adults with AIH-cirrhosis evaluated at Cedars-Sinai. Patients were categorized based on prednisone exposure: (≥7.5 mg/day for ≥6 months following cirrhosis diagnosis) exposure vs subthreshold exposure (with or without alternative therapies). The primary outcome was the HCC incidence rate and hazard ratio derived from Cox proportional hazards models. Secondary outcomes included HCC incidence using multivariable models adjusted per demographics and disease severity.

**Results:**

In unadjusted time-to-event analysis, prednisone use shortened HCC-free survival, the results indicated HCC developed in 25.4% of the prednisone group vs 9.7% of controls (*P* = .030). Multivariable analysis adjusted per age, gender, ethnicity, Child-Turcotte-Pugh, and alpha-fetoprotein confirmed prednisone as an independent risk factor (adjusted hazard ratio: 3.36; *P* = .040).

**Conclusion:**

The study findings suggested that prolonged prednisone exposure in patients with AIH-cirrhosis independently contributes to HCC development. This association appears to be supported by mechanistic pathways, driven by prednisone’s dose-dependent immunosuppressive and metabolic effects, disrupting immune surveillance, promoting hepatic fibrosis, and oncogenic pathways.

## Introduction

Autoimmune hepatitis (AIH) is a rare progressive inflammatory liver disease characterized by immune-mediated hepatocyte injury, elevated liver enzymes, and the presence of disease-specific autoantibodies.^1^ Two primary subtypes of AIH have been identified based on serologic markers: type 1 AIH and type 2 AIH, with the proposed type 3 AIH subtype as outlined in the 2025 European Association for the Study of the Liver (EASL) guidelines.^2^ Although its prevalence is relatively low, estimated at 10–20 cases per 100,000 individuals, AIH disproportionately affects females, with peak incidences occurring either in childhood/adolescence or after the age of 40.^1^, ^2^, ^3^ The insidious progression of AIH often results in chronic hepatic inflammation and fibrosis, with approximately half of untreated cases advancing to cirrhosis within a decade of diagnosis.^3^^,^^4^ Cirrhosis at the time of AIH diagnosis is associated with a significantly reduced 10-year survival rate of 61.9% as compared to 94.0% in patients without cirrhosis.^5^

The goal of treating AIH is to induce and maintain remission, alleviating symptoms, and halting or potentially reversing liver damage and fibrosis while minimizing adverse effects.^6^^,^^7^

Prednisone is a synthetic glucocorticoid derived from cortisone widely used for its anti-inflammatory and immunosuppressive properties. Prednisone remains the mainstay treatment for several autoimmune including AIH and chronic inflammatory conditions as the 30th most prescribed medication in the United States due to its broad immunosuppresive effects.^8^ The American Association for the Study of Liver Diseases (AASLD) recommends prednisone either as monotherapy or in combination with azathioprine. The combination therapy is preferred to minimize long-term corticosteroid toxicity.^9^ Prednisone’s prolonged use is linked to significant adverse effects, including metabolic disturbances, osteoporosis, and immunosuppression. The addition of steroid-sparing therapy such as mycophenolate mofetil and tacrolimus helps to alleviate the dependence on prednisone therapy. Such agents also exert more unique immunomodulatory effects with an acceptable safety profile leading to safe withdrawal of prednisone.^10^, ^11^, ^12^

Uncontrolled AIH promotes progressive fibrosis, often culminating in cirrhosis—a critical risk factor for hepatocellular carcinoma (HCC), with annual incidence rates ranging from 1% to 2%, depending on the underlying liver disease etiology.^13^

Cirrhosis resulting from AIH is recognized as a potential precursor to malignancy in the context of prolonged disease duration and incomplete biochemical control.^14^

The pathogenesis of HCC in AIH-related cirrhosis involves a multifactorial interplay of chronic immune activation, oxidative stress, hepatocyte regeneration, and fibrotic remodeling, all of which contribute to genomic instability and potential malignant transformation.^15^ The pro-inflammatory environment creates conditions conducive to the accumulation of DNA mutations and oncogenic signaling pathway activation. A recent meta-analysis of 16 studies examined HCC incidence in patients with AIH-related cirrhosis and had a pooled HCC incidence of 10.07 per 1000 person-years, with nearly all HCC cases occurring in patients with cirrhosis.^14^ As a result, current EASL guidelines recommend routine HCC surveillance in cirrhotic patients with AIH, consistent with protocols used for other chronic liver disease etiologies.^2^

Emerging evidence suggests that chronic glucocorticoid exposure may contribute to carcinogenesis, particularly increasing the risk of HCC.^16^ A population-based cohort study in South Korea found that long-term glucocorticoid use was associated with a 1.23-fold increased risk of cancer, with a more pronounced risk for liver and lung cancer.^17^

The relationship between prednisone and carcinogenesis is complex and context dependent; while prednisone exerts antiproliferative effects on certain malignancies, other studies have suggested that prolonged glucocorticoid therapy may exert oncogenic effects, contributing to an elevated cancer risk.^16^, ^17^, ^18^ Prolonged corticosteroid use can induce metabolic dysfunction, which may contribute to liver cirrhosis.^19^ Chronic immunosuppression can create an environment conducive to oncogenesis by impairing immune surveillance.^20^ A study using a representative claims database established from the Taiwan National Health Insurance Program found that oral corticosteroid use in patients with chronic liver disease and diabetes significantly increased the risk of HCC.^21^ Additionally, previous research highlights glucocorticoids as having dual roles within the pretumor microenvironment: initially providing protective effects but subsequently, with chronic exposure, potentially promoting carcinogenesis.^16^

Extracting from these studies, there is a clear need for further research into the oncogenic risks of prolonged prednisone use, particularly in patients with AIH and cirrhosis; as a result, we conducted a study to evaluate whether long-term prednisone use in patients with AIH cirrhosis is associated with an increased risk of HCC.

## Methods

### Cohort Study

#### Study design

We conducted a retrospective observational study that included 121 patients with AIH-related cirrhosis who were initially evaluated between January 2001 and December 2023. Clinical and demographic data, and immunomodulator use, were collected from electronic medical records using data from the Cedars-Sinai database, identified through the DEEP6 Cohort Builder technology. Ethical approval for this study was granted by the Ethics Committee of Cedars-Sinai IRB (STUDY00004026). Our study was conducted in accordance with the Strengthening the Reporting of Observational Studies in Epidemiology (STROBE) guidelines and checklist for cohort studies.^22^

#### Study population

##### Inclusion criteria

Patients were followed from the index cirrhosis diagnosis until HCC, liver transplantation, or death. Eligible patients had sufficient clinical documentation and medication history, enabling the assessment of disease progression to HCC or for a minimum of 12 months. Patients were followed for up to the event, with analyses focused HCC diagnosis endpoint. Patients were stratified into two groups: the prednisone-exposed group, defined as patients who received prednisone therapy at a consistent dose of ≥7.5 mg/day for a minimum of 6 months following the diagnosis of cirrhosis; and the subthreshold group, who received subthreshold prednisone with or without different immunomodulator therapies. Prednisone exposure was classified as prolonged moderate dose if the mean daily dose was ≥ 7.5 mg prednisone maintained for at least six consecutive months after the diagnosis of cirrhosis. The 7.5-mg threshold corresponds to the lowest “maintenance” dose at which tapering is no longer routinely recommended, as stated in both AASLD and EASL guidance.^2^^,^^9^ A 6-month duration was chosen a priori to capture the period over which sustained glucocorticoid-driven metabolic and immunologic alterations emerge. A binary “stopped vs continued” analysis was not performed because the ability to discontinue steroids is contingent upon an early biochemical response, thereby introducing indication bias.^23^

We define steroid use as subthreshold for patients who briefly (<6 months) received prednisone solely during the initial disease stabilization, as these short-term exposures are unlikely to produce meaningful chronic immunological and metabolic effects relevant to HCC risk. The chosen dose (≥7.5 mg/day) and duration (≥6 months) thresholds align with clinical guidelines for chronic AIH management.^23^, ^24^, ^25^ Patients treated exclusively with initial induction therapy with or without receiving other immunomodulatory therapies such as including azathioprine or budesonide were classified under the subthreshold group. We sought to include biochemical remission, defined per AASLD/EASL criteria as simultaneous normalization of alanine aminotransferase and total immunoglobulin G (IgG) within 12 months of cirrhosis diagnosis.^2^^,^^9^ However, IgG was missing in 63% of otherwise eligible charts, precluding reliable classification. To avoid misclassification bias and loss of power, biochemical remission was not retained as an analytic covariate; this limitation is addressed below.

##### Exclusion criteria

We excluded patients who never received any AIH therapy to minimize confounding by indication. To reduce confounding by concomitant immunosuppression or metabolic cofactors, we excluded patients who, at the time of cirrhosis diagnosis, were receiving systemic immunosuppressants or had an immunocompromised state unrelated to AIH (eg, solid-organ transplantation, HIV, active chemotherapy). Nearly all patients who received any therapy beyond prednisone were treated with azathioprine, employed as a steroid-sparing immunomodulator rather than a broad-spectrum immunosuppressant.^26^ Patients with cirrhosis of multifactorial etiology or cirrhosis-modifying comorbidities such as insulin-dependent diabetes mellitus or documented alcohol use disorder were also excluded. Patients diagnosed with cirrhosis of multifactorial etiology (eg, concurrent AIH combined with one or more of other liver diseases such as alcohol-related liver disease) were also excluded. Patients who were deceased, lost to follow-up, or underwent liver transplantation prior to HCC development were not excluded but were appropriately censored at the date of last follow-up or transplantation, respectively. A total of 121 patients met the final eligibility criteria for the study.

#### Outcomes design

The primary outcome was the incidence of HCC during follow-up and annual HCC incidence rates. Cox proportional hazard models, utilizing a standardized HCC surveillance protocols,^27^ comparing HCC development between prednisone-exposed and subthreshold patients.

Secondary outcomes included estimation of HCC incidence rate across groups and evaluation of risk factors for HCC with multivariable models with adjustments for gender, race, and age at cirrhosis diagnosis and severity of liver dysfunction as represented by Child-Turcotte-Pugh (CTP) classification and alpha-fetoprotein (AFP) levels. For this analysis, the severity of cirrhosis is classified based on the CTP score into 2 groups, CTP (5–7 points) and CTP (8–15 points).^28^ We chose not to incorporate the model for end-stage liver disease (MELD) score in our models as the primary outcome of interest was HCC risk rather than survival. MELD score is more predictive of mortality than oncogenic progression; additionally, MELD components—particularly serum sodium and creatinine—were inconsistently documented in our dataset, resulting in extreme or unreliable MELD values that compromised the integrity of the secondary outcome analyses.^28^^,^^29^ The CTP score was selected instead, as it remains a robust measure of baseline liver disease severity and was more reliably captured across our cohort.^30^

#### Statistical analysis

Descriptive statistics were used to summarize baseline demographic and clinical characteristics (Table 1). Continuous variables were reported as means and standard deviations or medians and interquartile ranges (IQRs), depending on data normality. Categorical variables were presented as frequencies and percentages. Baseline characteristics were compared between the prednisone and other groups using chi-square or Fisher’s exact tests for categorical variables and *t*-tests for continuous variables. Annual incidence rate was used to account for differences in follow-up duration between groups. To estimate the relative risk of HCC, a Cox proportional hazards model was used, both unadjusted and adjusted for gender, race, and age at cirrhosis diagnosis. Hazard ratios (HRs) with 95% confidence intervals (CIs) were reported to determine independent risk factors for HCC. All statistical analyses were conducted using R (version 4.4.2) or SPSS (version 29.0), and a two-tailed *P* value <.05 was considered statistically significant. We also conducted a sensitivity Kaplan–Meier analysis stratified HCC-free survival by cumulative prednisone duration (<12 months vs ≥ 12 months). Curves with 95% confidence bands and accompanying numbers-at-risk are presented in Figure A1.

**Table 1:** Baseline Characteristics and Primary Outcomes

Baseline metric	Prednisone-exposed (≥7.5 mg × ≥ 6 mo)	Unexposed/Subthreshold	*P* value
Sample size (AIH + cirrhosis)	59	62	—
Cumulative prednisone duration, mo, median (IQR)	31 mo (IQR 13–60)	1 mo (IQR 1–63)	*P* < .001
Age, median (IQR) (y)	54.0 [33.0–63.0]	55.0 [48.2–67.8]	*P* = .16
Male sex	12 (20.3%)	8 (12.9%)	*P* = .33
White non-Hispanic	19 (15.7%)	27 (22.3%)	*P* = .47
White Hispanic	22 (18.2%)	24 (19.8%)	*P* = .85
Black	8 (6.6%)	3 (2.5%)	*P* = .11
Asian	7 (5.8%)	5 (4.1%)	*P* = .38
Other	1 (0.8%)	1 (0.8%)	*P* = 1.00
CTP ≥ 8	15 (25.4%)	14 (22.6%)	*P* = .83
AFP ≥ 20 ng/mL	5 (8.5%)	4 (6.5%)	*P* = .74
Analysis metrics			
HCC cases	15	6	—
Cumulative HCC incidence	**25.4%** (15/59)	9.7% (6/62)	***P* = .03** (χ^2^/Fisher)
Person-y of follow-up	453.4	557.4	—
Annual HCC incidence rate	**3.31/100 PY** (15/453.4)	0.90/100 PY (5/557.4)	—
Incidence rate ratio	—	—	**3.69** (95% CI 1.34–10.15), ***P* = .02**
Kaplan-Meier (HCC-free survival)	Shorter time to HCC	Longer time to HCC	Log-rank ***P* = .02**

Patients meeting the high-dose/long-term prednisone threshold had roughly a three-fold higher risk of developing HCC, both in cumulative terms and when expressed as incidence per person-year, with significantly shorter HCC-free survival on Kaplan-Meier analysis.

Bold values indicate statistical significance at *P* < .05.

PY, person-years.

## Results

### Primary Outcomes

**Figure 1:**
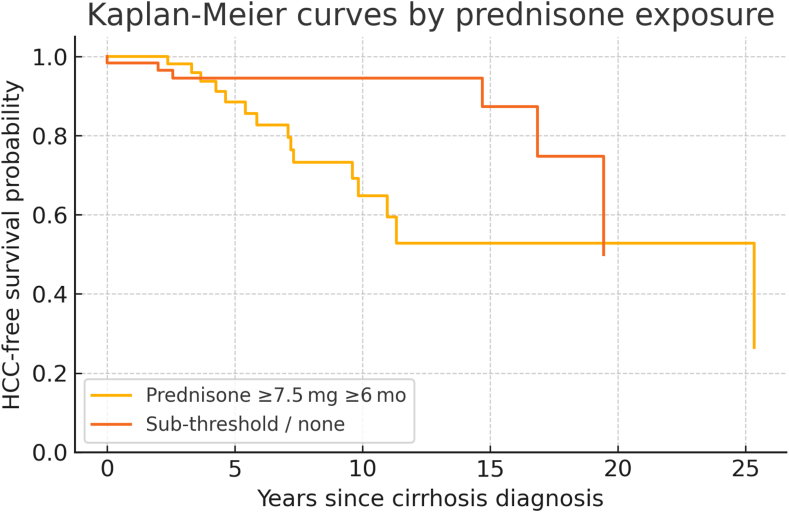
Kaplan-Meier plot comparing HCC-free survival for threshold prednisone versus subthreshold prednisone patients characteristics.

A total of 121 patients with AIH and cirrhosis were included, with 59 patients meeting the prednisone-exposed criteria and 62 subthreshold patients. Overall, the cohort accrued 684 person-years of observation, with a median follow-up of 6.4 years (IQR: 3.1–9.8). Median cumulative prednisone exposure was 31 months (IQR: 13–60) in the prolonged-exposure group versus 1 month (IQR: 1–63) in the subthreshold group (*P* < .001). The cumulative incidence of HCC was higher in prednisone-exposed patients 25.42% compared to subthreshold patients 9.67% achieving a statistical significance (*P* = .030). Annual HCC incidence rates were 3.31% in the prednisone-exposed group and 0.9% per year in the unexposed group. Kaplan-Meier survival analysis demonstrated a significantly shorter mean time to HCC development in the threshold prednisone group compared to the subthreshold prednisone group (Figure 1) with an annual incidence rate ratio of 3.69 (*P* = .0180) (Table 1).

### Secondary Outcomes

In the adjusted Cox regression model accounting for gender, race, age at cirrhosis diagnosis, CTP scores, and AFP, the prednisone-exposed group remained independently associated with an increased risk of HCC development (HR: 3.36; *P* = .040) (Table 2).

**Table 2:** Secondary Outcomes

Predictor (multivariable model)	Adjusted HR	95% CI	*P* value
Prednisone ≥ 7.5 mg × ≥ 6 mo (vs subthreshold/none)	**3.36**	1.06–10.66	**.040**
Male sex (vs female)	**4.95**	1.41–17.35	**.012**
Ethnicity-White Hispanic (vs White non-Hispanic)	**5.26**	1.25–22.08	**.023**
Ethnicity-Black	1.49	0.26–8.50	.656
Ethnicity-Asian	0.60	0.11–3.37	.559
Ethnicity-others^a^	—	—	—b
Age at cirrhosis diagnosis (per y)	1.04	1.00–1.08	.066
Child-Pugh ≥ 8 (vs ≤ 7)	0.46	0.10–2.05	.308
AFP ≥ 20 ng/mL (vs < 20)	3.87	0.46–32.83	.215

High-dose/long-term prednisone, male sex, and Hispanic ethnicity remain independent predictors of HCC after multivariable adjusted Cox model. Significant predictors of HCC were threshold prednisone exposure, male sex, and Hispanic ethnicity. CTP ≥ 8 shows a nonsignificant trend toward lower hazard (likely limited by small numbers in very advanced classes), increasing age and AFP ≥ 20 ng/mL suggest higher risk but did not reach statistical significance in this cohort.

Bold values indicate statistical significance at *P* < .05.

a “Other” race category had no HCC events and was omitted to avoid model instability.

b Parameter not estimable because of zero cases.

Patients with CTP ≥ 8 exhibited a decreased HR as compared to the CTP ≤ 7 group, but the association did not reach statistically significant (HR: 0.46; *P* = .308). Similarly, AFP levels of ≥20 ng/mL exhibited an increased HR (HR: 3.87; *P* = .215), likely due to sample size limitations, missing values for AFP, and small representation in CTP 10 and above.

The median age at cirrhosis diagnosis was relatively similar between the threshold prednisone group and the subthreshold prednisone group (54.0 vs 55.0 years; *P* = .16), exhibited a slight increased HR prednisone-exposed group with no significant differences observed in age distribution (HR: 1.04; *P* = .066) (Table 2).

Notably, male gender (HR: 4.95; *P* = .012) and Hispanic race (HR: 5.26; *P* = .023) were independently associated with elevated HCC risk, reinforcing prior studies suggesting an increased HCC risk among male patients with AIH and within Hispanic populations. As illustrated in Figure A1 and Table A1, patients exposed to prednisone for ≥ 12 months experienced a significantly higher HCC incidence than those treated for < 12 months (log-rank *P* = .022; adjusted HR: 3.18; 95% CI: 1.11–9.07).

## Discussion

Existing literature demonstrates that prednisone-induced metabolic dysregulation, including hepatic steatosis, insulin resistance, and the activation of oncogenic pathways significantly enhances the risk of fibrosis and tumorigenesis. Glucocorticoids like prednisone exacerbate this risk through their immunosuppressive effects, which impair immunosurveillance and alter the tumor microenvironment. Specifically, prednisone modulates tumor-associated macrophages and dendritic cells, shifting the immune balance toward an anti-inflammatory state.^16^ This suppresses cytotoxic CD8+ T cell activation and reduces the production of pro-inflammatory cytokines, thereby dampening antitumor immunity.^23^^,^^31^ As a result, hepatocytes with pre-existing mutations can evade immune detection and proliferate unchecked, further driving the progression of liver fibrosis and HCC. The activation of oncogenic pathways, such as phosphoinositide 3-kinase/Akt and Wnt/β-catenin, plays a critical role in this process. These pathways foster a pro-tumorigenic environment by enhancing cell survival, proliferation, and metabolic reprogramming.^32^^,^^33^ In addition, the immunosuppressive effects of glucocorticoids intensify these risks by disrupting immune surveillance and enabling malignant cells to evade detection and proliferate,^16^ the oncogenic potential of long-term prednisone use may be attributed to its complex impact on hepatic regeneration, metabolic dysfunctions, and antitumor immune surveillance suppression. Although glucocorticoid signaling initially helps suppress pro-neoplastic inflammation and reduce tissue damage, prolonged exposure can impair liver regeneration by hindering collagen deposition, growth factor expression, and vascularization.^34^ Over time, these disturbances foster a microenvironment that promotes hepatocyte proliferation and elevates the risk of malignant transformation.^35^^,^^36^

Our study reveals a significant association between prolonged prednisone use and an increased risk of HCC in patients with AIH-related cirrhosis. Prednisone, while essential to AIH management, may exert independent oncogenic effects when administered in the cirrhotic setting due to its immunosuppressive and metabolic influence.^37^ To isolate this relationship, our inclusion criteria required confirmed cirrhosis at baseline, ensuring that our findings reflect risk modulation in a population already predisposed to HCC due to hepatic fibrosis.^38^^,^^39^

**Figure 2:**
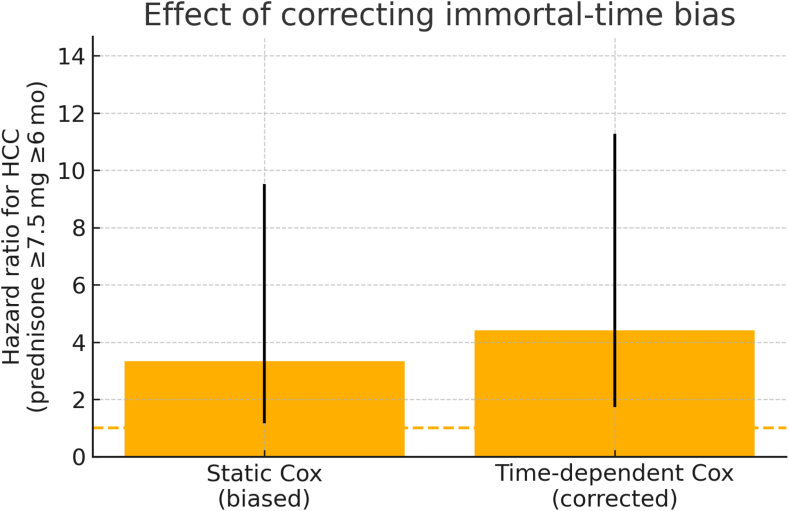
Extended Cox model addressing immortal-time bias for the estimated effect of prednisone on HCC risk. Static Cox (biased)—naïve model that classifies patients as “exposed” from time 0, HR ≈ 3.34 (95% CI: 1.17–9.52). Time-dependent Cox (corrected)—extended model that starts accruing exposed person-time only after the 6-month threshold, HR ≈ 4.41 (95% CI: 1.72–11.28).

Patients in the prednisone-exposed group exhibited a markedly higher HCC incidence cumulative and annual incidence rates compared to the subthreshold group, suggesting a potential carcinogenic risk of prolonged corticosteroid use. The reported 25.4% incidence reflects the cumulative proportion of patients in the threshold prednisone patients who developed HCC over the total follow-up period. Because prednisone exposure was defined as a ≥7.5 mg/day dose sustained for at least 6 months, there is an inherent induction period during which future exposed patients are still accumulating the requisite dose and cannot contribute events to the exposed risk set. Consequently, the Kaplan–Meier curves remain superimposed for ∼3 years before diverging. When prednisone was treated as a time-dependent covariate in an extended Cox model, the HR for HCC became attenuated in the first 2 years HR ≈ 3.34 (95% CI: 1.17–9.52) but increased significantly thereafter HR ≈ 4.41 (95% CI: 1.72–11.28), consistent with a latency period for glucocorticoid-driven tumorigenesis (Figure 2).

While AFP initially identified as a marker for HCC, elevated AFP levels may be associated with active liver injury and repair mechanisms.^40^ Despite its limitations, AFP continues to serve as a practical and widely adopted biomarker in assessing liver disease progression and surveillance for HCC.^30^^,^^41^^,^^42^ Current literature identifying AFP ≥ 20 ng/mL as a surrogate for malignant transformation in cirrhotic livers,^43^ serves as a surveillance marker in conjunction with imaging clinically.^28^ In contrast, the CTP classification estimates hepatic reserve and prognosis. CTP classification assesses the severity of cirrhosis and hepatic functional reserve based on clinical perimeters.^44^ While the CTP score includes subjective components sensitive to clinical interpretation, it remains a broadly utilized indicator of liver disease severity. Its consistent and reliable documentation within our cohort and inability to calculate a MELD score based on the available data justified its selection as the preferred measure to adjust for our multivariable models.^28^^,^^45^

Although AIH chiefly affects women, our analysis shows sustained prednisone raises HCC risk irrespective of sex. National Inpatient Sample data from 550,000 cirrhosis showed men experience more decompensation (38.8% vs 34%, *P* < .001) ,^46^ suggesting a more aggressive trajectory that may amplify their HCC susceptibility. Liang et al. likewise found male patients faced higher recurrence risk (HR: 1.48) and earlier relapse (odds ratio: 1.86) after resection.^47^ Thus, rigorous HCC surveillance is warranted for all prednisone-treated AIH cirrhosis, regardless of gender. Disparities extend to ethnicity: Hispanics often present with more advanced cirrhosis,^48^ mirroring our finding of a five-fold adjusted HCC hazard in this group (Table 2) and underscoring the need to explore genetic, environmental, and access-to-care drivers. Colapietro et al. reported only 1.7% overall HCC incidence among 1428 heterogeneous AIH patients, most noncirrhotic, rising to 6.6% at 30 years.^49^ Our cohort, confined to established cirrhosis and free of variant syndromes, displayed a markedly higher annual incidence (3.31% vs 0.90% with subthreshold prednisone) against the 1%–2% expected in broader cirrhosis populations,^50^ highlighting the synergistic oncogenic impact of advanced fibrosis and prolonged glucocorticoid immunosuppression.

### Limitations

These findings are limited by the retrospective design, which is vulnerable to selection, information, and residual-confounding bias. Despite adjustment for core demographics, unmeasured influences—including metabolic comorbidities, AIH subtype, exact corticosteroid dose-duration, treatment adherence, and lifestyle factors—may distort risk estimates. The relatively small sample size (N = 121) limits the precision of our estimates and reduces external generalizability. Because IgG measurements were unavailable for the majority of patients, biochemical response status could not be robustly incorporated; nevertheless, our stringent exclusion criteria for alternative immunosuppression, immunocompromised states, and high-impact metabolic comorbidities mitigate the principal nonsteroid determinants of persistent inflammatory activity. Moreover, the study demonstrates association, not causation, between prednisone exposure and HCC. Prospective analyses that disentangle the independent oncogenic effects of specific immunomodulators (azathioprine, budesonide, mycophenolate, and tacrolimus) and combination regimens are needed.

## Conclusion

Our results show that long-term prednisone exposure in patients with AIH-related cirrhosis is independently associated with a significantly increased risk of HCC. This association calls for further mechanistic studies to elucidate prednisone’s role in liver carcinogenesis and supports the development of evidence-based guidelines for optimized corticosteroid use and cancer surveillance in susceptible individuals. These results should be interpreted within the broader context of the effect of the immunosuppressive management in chronic liver disease. Prednisone remains an integral component of AIH therapy and can lead to histologic remission across a range of clinical settings. Our current analysis aimed to study the risk of cumulative corticosteroid exposure in the patients with AIH cirrhosis. Further investigation through larger, multicenter studies is warranted to validate our observations and improve our understanding of the effect of immunosuppressive exposure and hepatocarcinogenesis.

## Supplementary Material

Tables and fgure legend
