# Supplementary material for: Long-Term Prednisone Use Increases Hepatocellular Carcinoma Risk in Autoimmune Hepatitis Cirrhosis: A Retrospective Cohort Study
Source: Gastro Hep Adv. 2025 Aug 30;4(10):100784. doi: 10.1016/j.gastha.2025.100784 (PMC12547222; doi:10.1016/j.gastha.2025.100784)
Supplement: Tables and fgure legend [file mmc1.docx]

**Table S1-** HCC-free survival by cumulative prednisone duration (< 12 months vs ≥ 12 months).

| **Years** | **Patients at risk (≥ 12 mo)** | **Patients at risk (< 12 mo)** |
| --- | --- | --- |
| 0 | 66 | 55 |
| 2 | 65 | 42 |
| 4 | 50 | 36 |
| 6 | 41 | 25 |
| 8 | 33 | 19 |
| 10 | 26 | 13 |

Supplementary Table S1 patients exposed to prednisone for ≥ 12 months experienced a significantly higher HCC incidence than those treated for < 12 months (log-rank p = 0.022; adjusted HR 3.18, 95 % CI 1.11–9.07).

**Figure S1**- A sensitivity Kaplan–Meier analysis stratified HCC-free survival by cumulative prednisone duration (< 12 months vs ≥ 12 months).
