# Supplementary figures and images for: Long-Term Prednisone Use Increases Hepatocellular Carcinoma Risk in Autoimmune Hepatitis Cirrhosis: A Retrospective Cohort Study
Source: Gastro Hep Adv. 2025 Aug 30;4(10):100784. doi: 10.1016/j.gastha.2025.100784 (PMC12547222; doi:10.1016/j.gastha.2025.100784)

# Kaplan-Meier Curves: Prednisone Duration and HCC

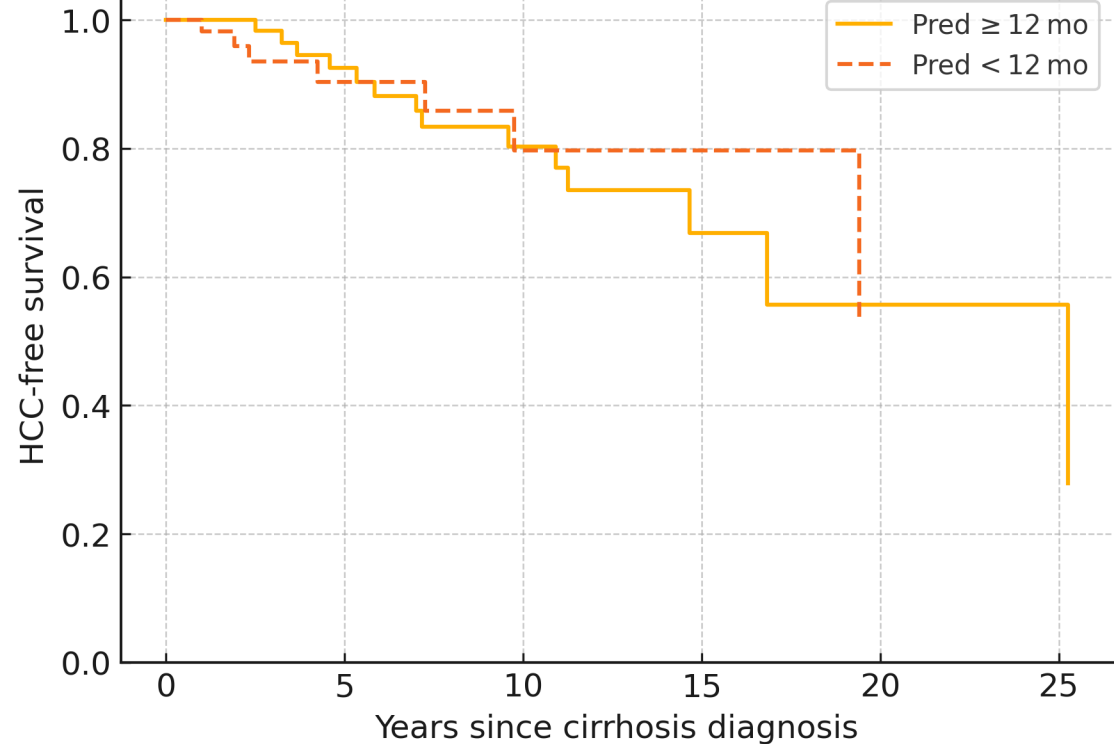

Supplement: Figure S1 [file mmc2.pdf]
